# Increased expression of calponin 2 is a positive prognostic factor in pancreatic ductal adenocarcinoma

**DOI:** 10.18632/oncotarget.17701

**Published:** 2017-05-09

**Authors:** Zhaoyan Qiu, Yi Chu, Bing Xu, Qian Wang, Mingzuo Jiang, Xiaowei Li, Gang Wang, Pengfei Yu, Guoxiao Liu, Hua Wang, Huijie Kang, Jiayu Liu, Yu Zhang, Jian-Ping Jin, Kaichun Wu, Jie Liang

**Affiliations:** ^1^ Department of General Surgery, Chinese PLA General Hospital, Beijing, China; ^2^ State Key Laboratory of Cancer Biology and Institute of Digestive Diseases, Xijing Hospital, The Fourth Military Medical University, Xi’an, China; ^3^ Department of General Surgery, Tangdu Hospital, The Fourth Military Medical University, Xi’an, China; ^4^ Department of Molecular Cellular and Developmental Biology, Yale University, New Haven, CT, USA; ^5^ Department of Cardiovascular Surgery, General Hospital of Lanzhou Military Area Command, Lanzhou, China; ^6^ Department of Physiology, Wayne State University School of Medicine, Detroit, MI, USA

**Keywords:** calponin 2, PDAC, prognosis, immunohistochemistry, cell transfection

## Abstract

Calponin 2 plays an important role in regulating actin cytoskeleton, which is critical for cell division and migration. Previous studies have demonstrated that calponin 2 inhibits prostate cancer cell proliferation and metastasis. However, the role of calponin 2 in pancreatic tumor growth, metastasis and patient survival remains unclear. Here, we demonstrate that the level of calponin 2 is a positive prognostic factor for patients with pancreatic ductal adenocarcinoma (PDAC). Patients with high calponin 2 expression in the tumor presented less lymph node metastasis and longer survival. Knockdown of calponin 2 facilitated pancreatic cancer cell proliferation and metastasis. Further experiments suggested that PI3K/AKT, NF-κB, Vimentin, Fibronectin, Snail and Slug were upregulated and E-cadherin was downregulated after calponin 2 was knocked down, implicating altered functions in PDAC proliferation and metastasis. In addition, we verified that calponin 2 functioned through inhibiting PI3K/AKT and NF-κB pathways. Our study suggests that the upregulation of calponin 2 in PDAC correlates to lower malignancy and presents a novel target for the development of new treatment.

## INTRODUCTION

Pancreatic ductal adenocarcinoma (PDAC) is one of the most formidable malignancies. It is the fourth leading cause of cancer-related death worldwide with an overall 5-year survival rate < 5% [1, 2]. Only 10–15% patients are eligible for surgical resection due to metastasis and advanced tumor-stage at the time of diagnosis [3]. For those unresectable cases, treatment with chemoradiation has a median survival time of less than one year [4, 5]. Considering the high mortality rate of PDAC and poor patient response to current therapies, the development of new and effective treatment strategies for PDAC is urgently needed.

Calponin is an actin cytoskeleton-associated regulatory protein, which has three isoforms in vertebrates: calponin 1, calpoin 2 and calponin 3 [6–8]. Calponin 1 is expressed in smooth muscle and plays a role in regulating contractility. Calponin 3 is expressed in smooth muscle and nonmuscle cells such as trophoblasts, myoblasts and B lymphocytes. In comparison, calponin 2 has a broader tissue distribution to express in smooth muscle, epithelial cells, fibroblasts, lung alveolar cells, endothelial cells and white blood cells [6, 9–11], and plays a role in regulating cell proliferation and motility [6]. These characteristics make calponin 2 a likely player in tumor development. Calponin 1 has been investigated in various tumors and identified as a protective factor in melanoma, ovarian cancer, renal angiomyolipoma and colon cancer [12–15]. Despite the wide distribution of calponin 2 in a variety of tissue types, its role in cancer biology has only been elucidated in the inhibition of prostate cancer metastasis [16].

Given that calponin 2 is expressed in various cell types including epithelial cells and inhibits cell proliferation and migration [6], we investigated calponin 2 expression in PDAC tumor tissues for potential roles in the development of PDAC. In the present study, we compared the expression of calponin 2 in PDAC tissues and non-tumor tissues from 119 patients and analyzed the correlation between the level of calponin 2 expression and clinicopathological parameters and survival. Furthermore, we investigated the function of calponin 2 in cancer cell proliferation and metastasis using two PDAC cell lines. We also explored two signaling pathways that are significantly changed after the knockdown of calponin 2 expression.

## RESULTS

### Expression of calponin 2 is increased in PDAC tumor tissues and correlated with clinicopathological parameters and patient survival

**Figure 1:**
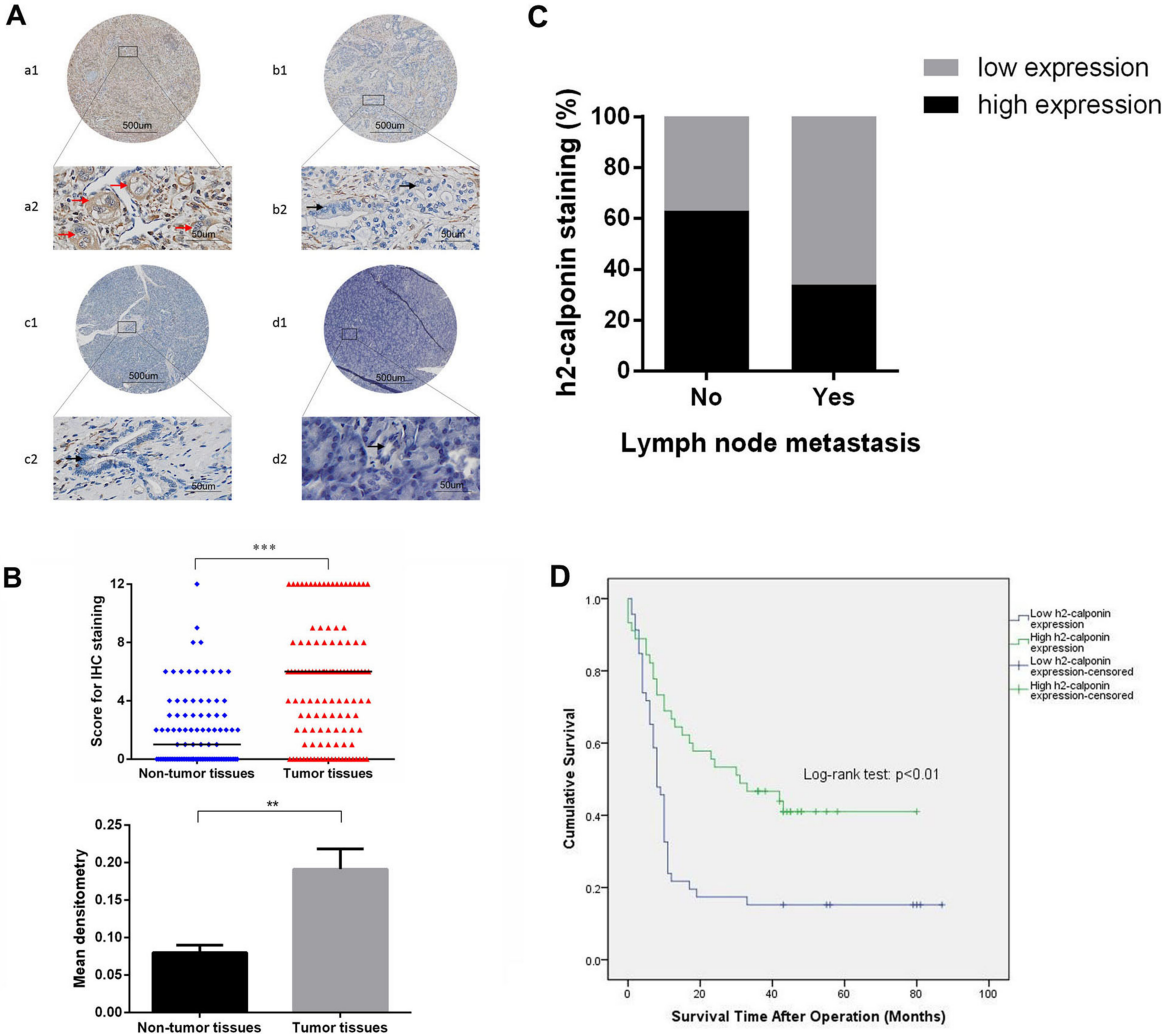
Expression of calponin 2 in PDAC tumor tissues and adjacent non-tumor tissues (**A**) Representative IHC staining of PDAC tissues, adjacent non-tumor tissues and normal pancreatic tissues. a1 and a2 represent high expression (red arrows) of calponin 2 in PDAC samples. b1 and b2 are low (or no) expression (black arrows) of calponin 2 in PDAC samples. c1, c2 and d1, d2 represent low (or no) expression of calponin 2 in pancreatic ductal cells (black arrows) in adjacent non-tumor tissues and normal tissues respectively. Some other cell types were also positive, such as endothelial cells and immune cells. Whole slide staining was performed in all samples (a1, b1, c1, d1, × 40 magnification) and a2, b2, c2, d2 show representative focal images (× 400 magnification). (**B**) Score for IHC staining between tumor tissues and adjacent non-tumor tissues, each bar represents the median, ***p < 0.001. (**C**) Higher percentage of patients with no lymph node metastasis have high calponin 2 expression than that of patients with positive lymph node metastasis. (**D**) Kaplan-Meier Survival Analysis shows that increased expression of calponin 2 is associated with better survival of PDAC patients (*P* < 0.01, log rank test). PDAC, pancreatic ductal adenocarcinoma; IHC, immunohistochemistry.

To determine the role of calponin 2 in PDAC, we first examined the expression of calponin 2 in PDAC and adjacent non-tumor tissues using immunohistochemistry. Calponin 2 was stained mainly in the cytoplasm (Figure 1A). Calponin 2 was highly expressed in 60 of the 119 tumor tissues (50.42%) and in 13 of the 99 non-tumor tissues (13.13%). The expression was significantly higher in tumor tissues than that in non-tumor tissues (*P <* 0.001, Table 1). Additionally, the score for IHC staining and mean densitometry of tumor tissues was significantly higher than that of non-tumor tissues (Figure 1B). Calponin 2 expression was negatively correlated with lymph node metastasis, but not with gender, age, location, differentiation, depth of invasion or TNM stage (Table 2). Patients without lymph node metastasis had a higher ratio (63%) of high calponin 2 expression than that of those with positive lymph node metastasis (34%) (Figure 1C). This finding indicates that calponin 2 may play an inhibitory role in lymph node metastasis.

**Table 1:** Comparison of calponin 2 expression between tumor tissues and adjacent non-tumor tissues

Tissue type	Calponin 2 expression		*P* value
	Low	high	
Cancer tissues	59	60	< 0.001
Adjacent non-cancer tissues	86	13	

**Table 2:** Association of calponin 2 expression with clinicopathological parameters of patients with PDAC

Parameter	*n*	Expression of calponin 2 (*n*)		*p*
		low	high	
Gender				0.066
Male	70	39	31	
Female	40	15	25	
Age				0.246
< 60	51	22	29	
≥ 60	59	32	27	
Location				0.171
Head or neck	67	29	38	
Body or tail	35	19	16	
Diffuse	8	6	2	
Differentiation				0.079
Well and moderate	74	32	42	
Poor	36	22	14	
Depth of invasion				0.081
T1–T2	86	46	40	
T3–T4	24	8	16	
Lymph node metastasis				< 0.01
No	63	23	40	
Yes	47	31	16	
TNM stage				0.091
I	46	17	29	
II	57	33	24	
IV	7	4	3	

To assess whether calponin 2 expression had an effect on the overall survival of PDAC patients, we performed Kaplan-Meier survival analysis on patients with complete survival information (91 cases). The patients with high calponin 2 expression had better prognosis than those with low expression (*P <* 0.01, Log-rank test, Figure 1D). Furthermore, we performed univariate and multivariate Cox regression analysis to examine whether calponin 2 expression was an independent prognostic factor for PDAC patients. The variables of gender, age, location, differentiation, depth of invasion, lymph node metastasis, TNM stage and calponin 2 expression were included in the analysis and the results showed that calponin 2 expression had statistically significant beneficial effect on survival *P <* 0.05 (Table 3).

**Table 3:** Univariate analysis of association between clinicopathological parameters and survival of PDAC patients

Parameters	*N*	HR	95% CI for HR	*P* value
Gender				
Male	55	1.195	0.722–1.980	0.489
Female	36			
Age				
< 60	44	0.881	0.542–1.434	0.611
≥ 60	47			
Location				0.823
Head or neck (reference)	56	1		
Body or tail	27	1.174	0.689–2.002	0.555
Diffuse	8	1.155	0.488–2.734	0.743
Differentiation				
Well and moderate	63	0.485	0.292–0.807	< 0.01
Poor	28			
Depth of invasion				
T1–T2	74	1.002	0.535–1.877	0.996
T3	17			
Lymph node metastasis				
No	53	0.462	0.281–0.759	< 0.01
Yes	38			
TNM stage				< 0.05
I (reference)	40	1		
II	49	2.183	1.292–3.688	< 0.01
IV	2	2.854	0.666–12.220	0.158
Calponin 2 expression				
Low	46	2.300	1.386–3.817	< 0.01
High	45			

Among the parameters considered, lymph node metastasis and TNM stage are confounding outcomes, of which TNM stage was included in multivariate analysis using the Enter method. In this test, calponin 2 expression and tumor differentiation state remain significantly different between groups (*P <* 0.05 and *P <* 0.01, respectively) (Table 4). These results demonstrate that calponin 2 expression is a beneficial prognostic factor for PDAC independent of the other factors.

**Table 4:** Multivariate analysis of correlation between clinicopathological parameters and survival of PDAC patients

Parameters	Coefficient	HR	95% CI for HR	*P* value
Differentiation (well and moderate vs. poor)	0.863	2.370	1.330–4.223	< 0.01
TNM stage				0.389
I (reference)		1		
II	0.486	1.626	0.689–3.835	0.267
IV	0.741	2.098	0.463–9.517	0.337
Calponin 2 expression (low vs. high)	−0.569	0.566	0.325–0.985	< 0.05

### Overexpression of calponin 2 inhibits PDAC cell proliferation and migration *in vitro*

**Figure 2:**
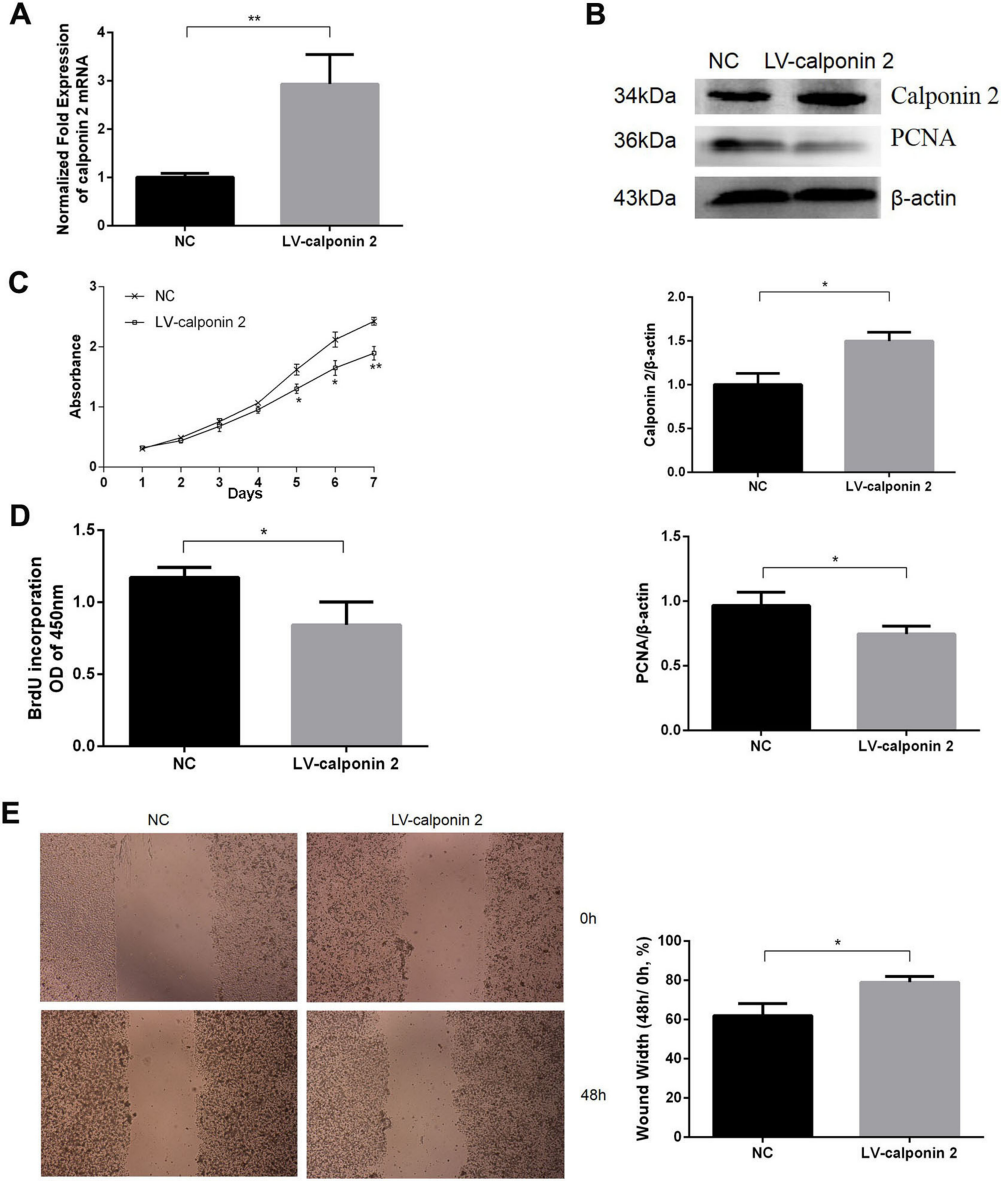
Overexpression of calponin 2 inhibits proliferation and migration of pancreatic cancer cells (**A**) Increased level of calponin 2 mRNA in lentivirus (LV)-calponin 2 infected cells compared with empty vector negative control (NC). (**B**) Increased protein level of calponin 2 and decreased protein level of PCNA in LV-calponin 2 infected cells is shown by WB and semiquantitative analysis (*P < 0.05). All gels were run under the same experimental conditions and repeated at least three times. (**C**) Decreased absorbance value and cell proliferation ability in LV-calponin 2 infected cells compared with NC (*P < 0.05, **P < 0.01). (**D**) BrdU assay showed that less BrdU was incorporated in LV-calponin 2 infected cells (*P < 0.05). (**E**) LV-calponin 2 infected cells migrates more slowly than that of NC (*p < 0.05). Magnification = × 40.

In calponin 2 stable transfected cell line (LV-calponin 2, MIA-PaCa2), PCR and WB detected that the expression of calponin 2 was increased compared with the vector transfected control (NC) (Figure 2A, 2B). The cell-proliferation marker PCNA (proliferating cell nuclear antigen) was downregulated after transfective overexpression of calponin 2 (Figure 2B). CCK-8 assay showed that overexpression of calponin 2 caused a decrease in cell proliferation (Figure 2C). BrdU assay showed that overexpression of calponin 2 decreased the BrdU incorporation (Figure 2D). *In vitro* wound healing assay indicated that the velocity of cell migration was significantly decreased after transfective overexpression of calponin 2 (Figure 2E). These data demonstrate that calponin 2 inhibits the growth and migration of PDAC tumor cells.

### Reduction of calponin 2 promotes PDAC cell proliferation and migration *in vitro*

**Figure 3:**
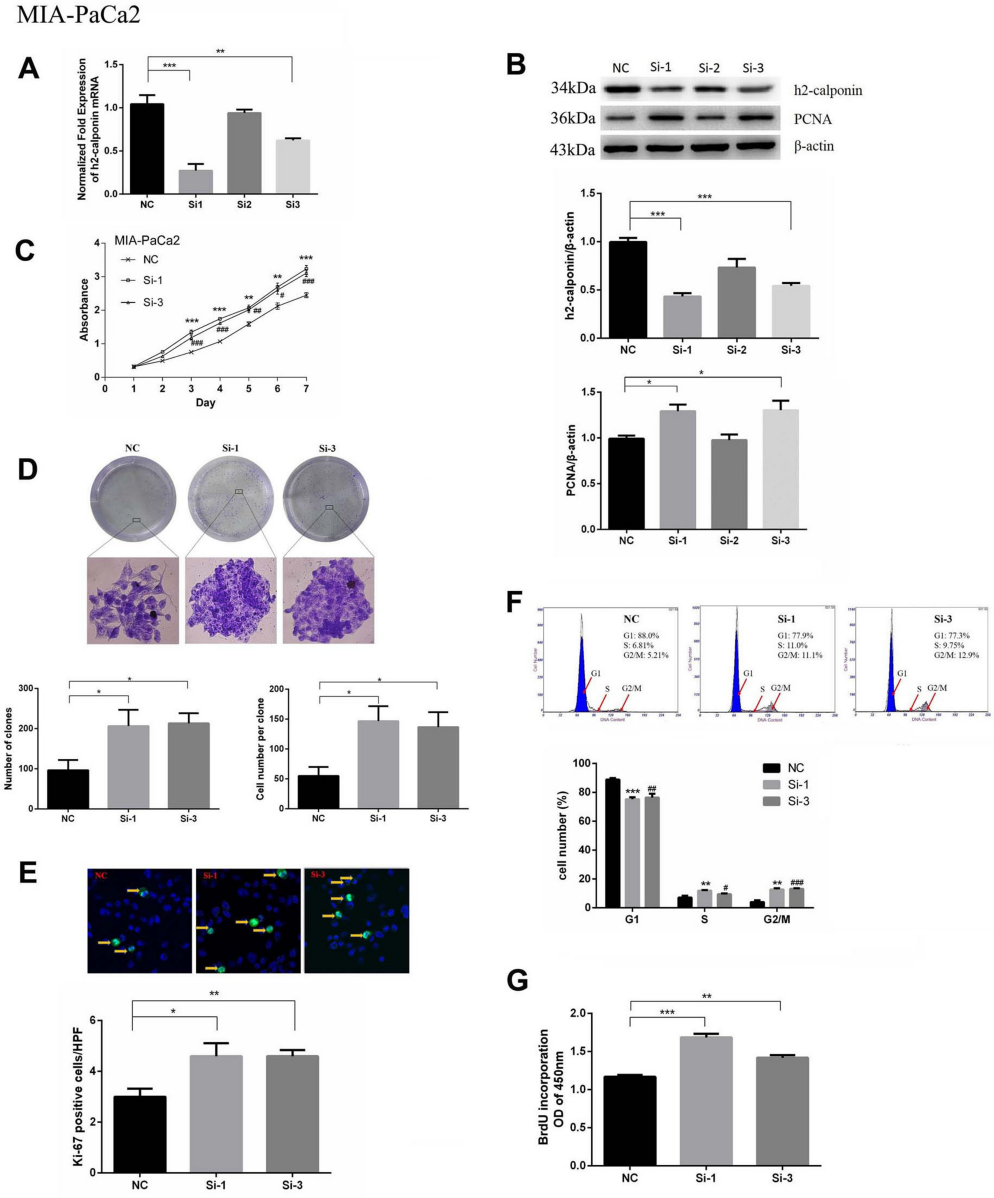
Knocking down calponin 2 accelerates proliferation of MIA-PaCa2 cells (**A**) Decreased level of calponin 2 mRNA in Si-1 and Si-3 cells as compared with NC (*P < 0.01, ***P < 0.001). (**B**) Decreased protein level of calponin 2 and increased protein level of PCNA in Si-1 and Si-3 cells shown by WB and semiquantitative analysis (*P < 0.05). All gels were run under the same experimental conditions and repeated at least three times. (**C**) Increased absorbance value and cell proliferation rate in Si-1 and Si-3 cells as compared with NC (*P < 0.01, ***P < 0.001, Si-1 versus NC; ^#^*P* < 0.05, ^##^*P* < 0.01, ^###^*P* < 0.001, Si-3 versus NC). (**D**) Increased colony formation in Si-1 and Si-3 cells as compared with NC. Magnification = ×100. (**E**) More Ki-67 positive cells (yellow arrows) in Si-1 and Si-3 cells compared with NC (*P < 0.05, *P < 0.01). Magnification = × 400. (**F**) More cells arrested in the S and G2/M stages in Si-1 and Si-3 cells as compared with NC (*P < 0.01, ***P < 0.001, Si-1 versus NC; ^#^*P* < 0.05, ^##^*P* < 0.01, ^#^*P* < 0.001, Si-3 versus NC). (**G**) BrdU assay showed that more BrdU was incorporated in Si-1 and Si-3 cells than that of NC (*P < 0.01, ***P < 0.001). NC, negative siRNA control; Si-1, calponin 2 siRNA1; Si-2, calponin 2 siRNA2; Si-3, calponin 2 siRNA3; PCNA, proliferating cell nuclear antigen.

**Figure 4:**
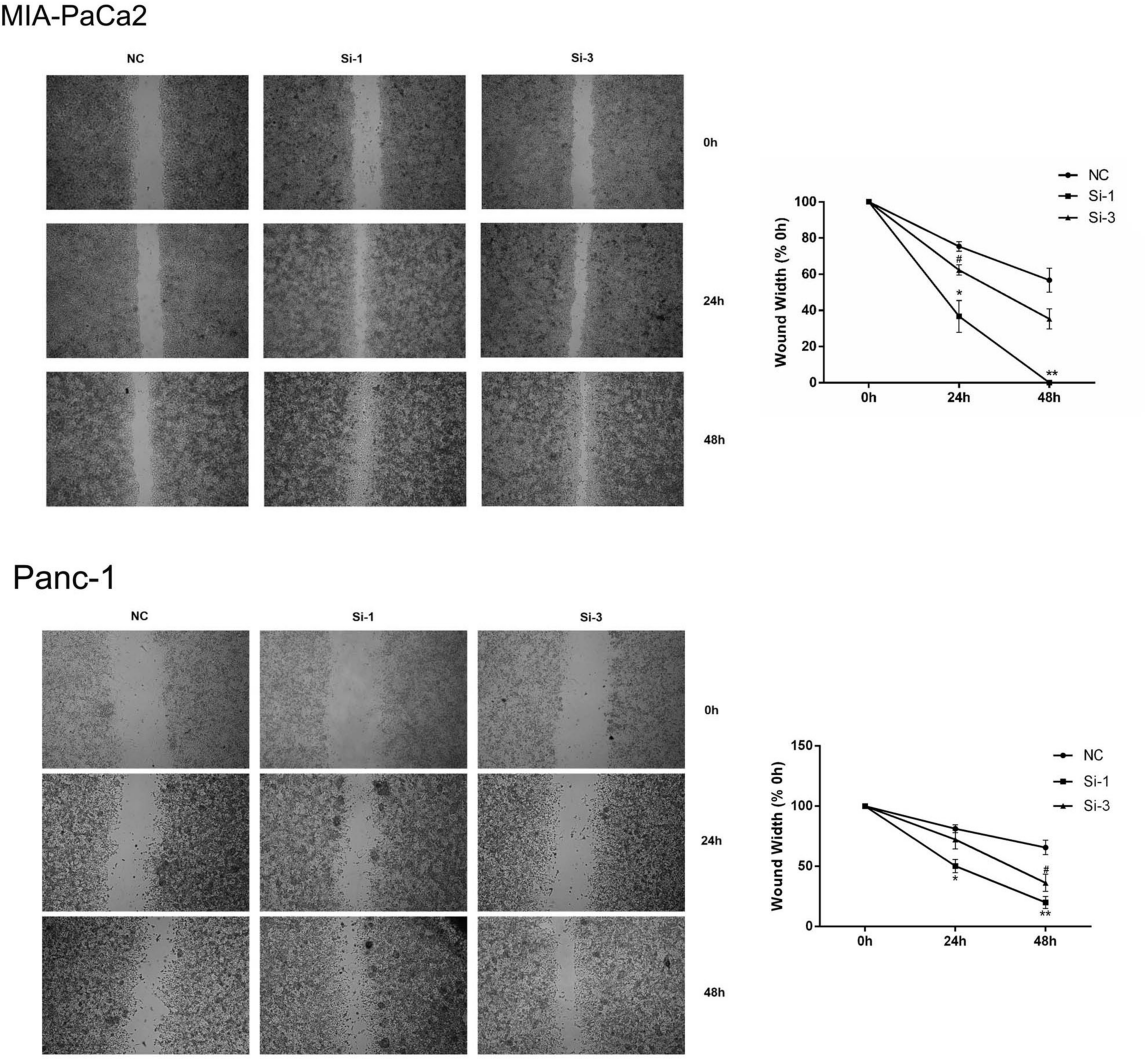
Silencing calponin 2 in MIA-PaCa2 and Panc-1 cells accelerates migration *In vitro* scratch wound healing assay, Si-1 and Si-3 cells migrates faster than that of NC (*P < 0.05, *P < 0.01, Si-1 versus NC; ^#^*P* < 0.05, Si-3 versus NC), magnification = × 40.

To further confirm the inhibitory function of calponin 2 in PDAC tumor cell proliferation and migration, we inhibited calponin 2 expression with RNA interference (RNAi) in the PDAC cell line MIA-PaCa2. After transfection with calponin 2 siRNA, cells were collected and analyzed with PCR and WB. Si-1 and Si-3 transfected cells had higher knockdown efficiency than that of Si-2 (Figure 3A, 3B). The cell-proliferation marker PCNA (proliferating cell nuclear antigen) was upregulated after calponin 2 knockdown (Figure 3B). In CCK-8 assay, reduction of calponin 2 by RNAi caused an increase in cell proliferation (Figure 3C). In the colony forming assay, reduced calponin 2 level increased colony formation and colony size (Figure 3D). Immunofluorescence staining showed that calponin 2 knockdown increased Ki-67 positive cells (Figure 3E). The proportion of cells in S and G2/M phage was higher in Si-1 and Si-3 transfected cells than that in NC (Figure 3F). BrdU assay showed that more BrdU was incorporated in Si-1 and Si-3 transfected cells than that of NC (Figure 3G). We also performed the experiments mentioned above on another PDAC cell line, Panc-1, and the results were consistent with that of MIA-PaCa2 (Supplementary Figure 1). *In vitro* wound healing assay was performed on MIA-PaCa2 and Panc-1. The results showed that the velocity of cell migration was significantly increased after calponin 2 knockdown (Figure 4). These data suggest that calponin 2 plays an inhibitory role in tumor cell proliferation and metastasis.

### Reduction of calponin 2 upregulates p-AKT and p-p65 and promotes EMT (Epithelial-mesenchymal transition) progress in PDAC cells

**Figure 5:**
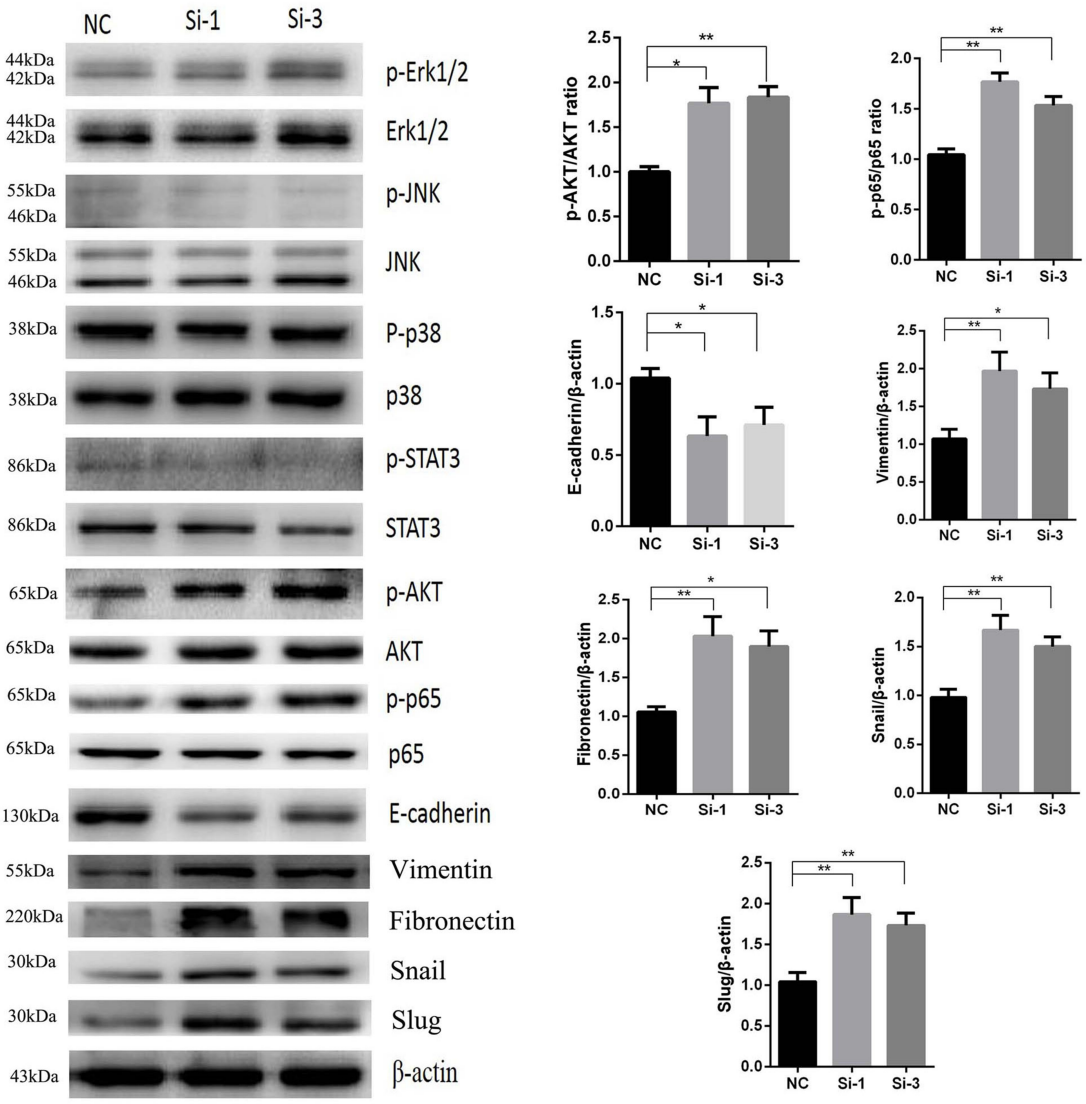
Increased protein levels of p-AKT, p-p65, vimentin, fibronectin, Snail, Slug and decreased protein level of E-cadherin in Si-1 and Si-3 cells compared with NC (*P < 0.05, **P < 0.01) No significant difference was detected in the levels of p-Erk1/2, p-JNK, p-p38 and p-STAT3. All gels were run under the same experimental conditions and repeated at least three times.

Although previous studies have investigated the role of calponin 2 in other tumors, the specific pathway by which calponin 2 functions has yet to be identified. To elucidate the pathway through which calponin 2 regulates cell proliferation and metastasis in PDAC, we examined protein expression of several canonical signaling pathway correlated with cell proliferation and metastasis. Since it is well documented that these pathways and EMT related proteins play a decisive role in PDAC development, we examined these pathways, including MAPK (p-Erk1/2/Erk1/2, p-p38/p38, p-JNK/JNK) [17], JAK-STAT (p-STAT3/STAT3) [18], NF-κB (p-p65/p65) [19], PI3K/AKT (p-AKT/AKT) [20] and EMT markers [21] such as E-cadherin, vimentin, fibronectin, Snail and Slug in MIA-PaCa2 to investigate the function of calponin 2 in PDAC. The results showed that P-AKT, p-p65, vimentin, fibronectin, Snail, Slug were upregulated and E-cadherin was downregulated, while other pathways exhibited no significant change after reduction of calponin 2 in MIA-PaCa2 cells (Figure 5).

### NF-κB and AKT pathways are involved in enhanced pancreatic cancer cell proliferation induced by calponin 2 knockdown

**Figure 6:**
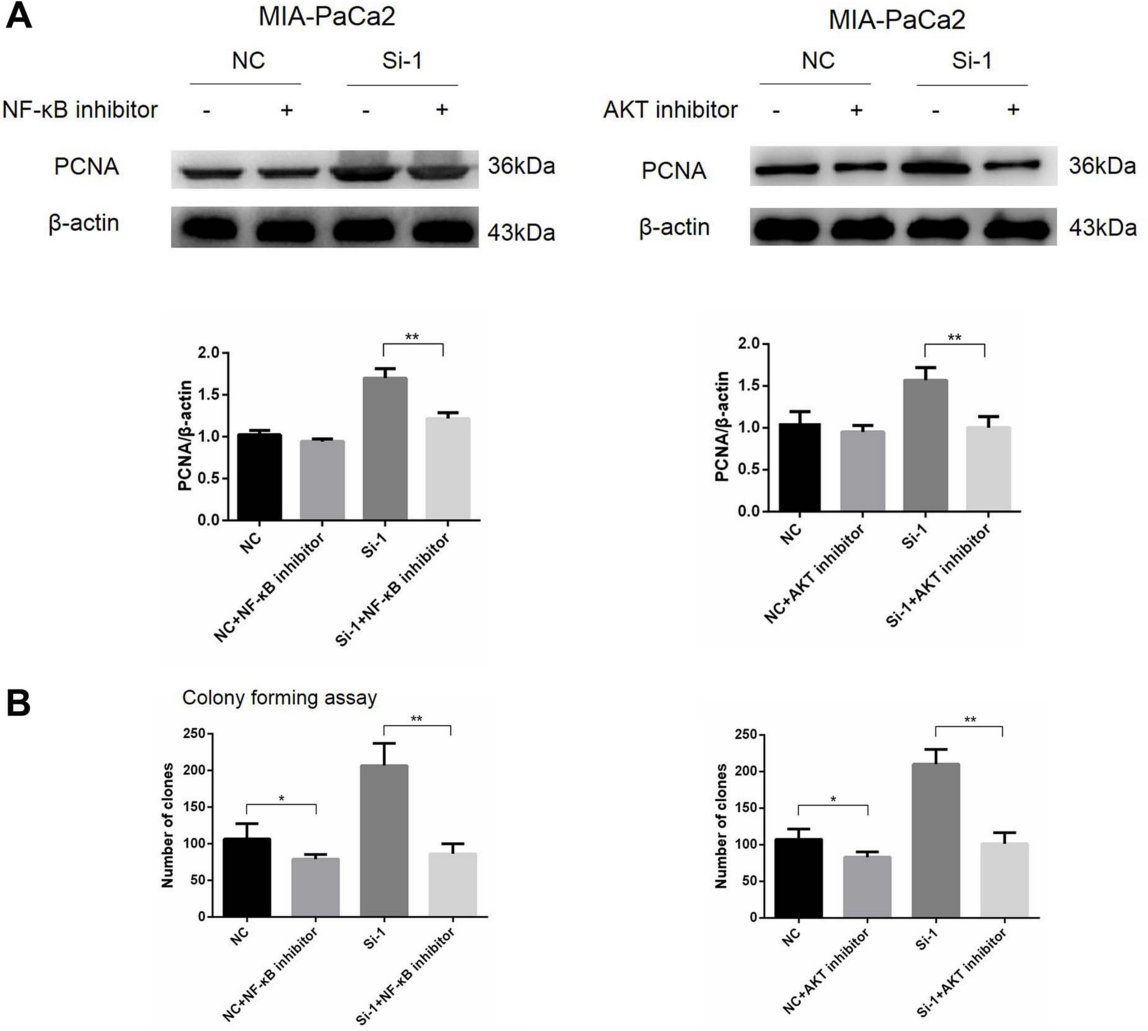
NF-κB and AKT signals promote cell proliferation in calponin 2-knockdwon pancreatic cancer cells (**A**) Protein expression of PCNA was significantly down-regulated in pancreatic cancer cells, more in calponin 2 knockdown cells (**P < 0.01). (B) The inhibition rate of colonies formation in calponin 2 knockdown cells was significantly higher than that of control cells after treatment of NF-κB and AKT inhibitors (*P < 0.05, **P < 0.01). All gels were run under the same experimental conditions and repeated at least three times.

To verify whether NF-κB and AKT pathways are involved in enhanced pancreatic cancer cell proliferation induced by calponin 2 knockdown, NF-κB and AKT specific inhibitors PDTC and LY294002 (Merck, 548000 and 440202) were used to treat calponin 2-knockdown MIA-PaCa2 cells and control cells. The concentration at 10 μM was used for resulting in cellular effects without significant cell death according to our preliminary experiments. After the inhibitor treatment for 48 h, protein expression of PCNA was significantly down-regulated in pancreatic cancer cells, more in calponin 2 knockdown cells (Figure 6A). The inhibition of colony formation in calponin 2 knockdown cells was significantly higher than that in control cells after the treatment with NF-κB and AKT inhibitors (Figure 6B).

## DISCUSSION

Despite progresses in cancer research, PDAC remains to be an incurable lethal disease with median survival time of less than one year [22]. It is difficult to detect the tumor at early stage. In most cases, PDAC has progressed to an advanced stage when diagnosed, too late for radical pancreatic resection [23]. Chemoradiation for PDAC is also no very effective [4, 5]. So it is necessary to seek new pharmacological targets for the treatment of PDAC.

Calponin 2 is an isoform of the calponin family with broad tissue distributions. Recent studies have demonstrated the role of calponin 2 in mechanoregulation and modulation of the actin cytoskeleton in response to the mechanical environment [6]. Since the actin cytoskeleton plays a central role in cell division and motility [24–26], calponin 2 plays a role in inhibiting cell proliferation and migration, which implicates an involvement in tumor genesis and progression [6, 27].

In our present study, we demonstrate that calponin 2 is a unique prognostic factor for patients with PDAC. Patients with high calponin 2 expression in tumor cells had more favorable survival rate than those with low expression. The expression of calponin 2 was negatively correlated with lymph node metastasis. Overexpression of calponin 2 inhibited PDAC tumor cells proliferation and migration, whereas reduction of calponin 2 in PDAC cells resulted in increased proliferation and migration rate. Calponin 2 is correlated to less tumor metastasis in patients with PDAC, and increased calponin 2 expression predicted a better prognosis. These findings are consistent with the results of our previous study [16], demonstrating that reduction of calponin 2 may facilitate prostate cancer cell proliferation, migration and metastasis to bone tissues.

The inhibitory effect of calponin 2 overexpression on proliferation and migration of pancreatic cancer cells was moderate. This may reflect that calponin 2 is an actin cytoskeleton-associated regulatory protein expressed in abundance in cells as compared with transcription factors or signaling proteins. In addition, we found that the activity of AKT and NF-κB pathways, which also regulate cell proliferation and metastasis [28, 29], was upregulated in PDAC cells. Inhibiting AKT and NF-κB pathways significantly reduced the proliferation of calponin 2-knockdown pancreatic cancer cells. E-cadherin, the loss of which contributed to an EMT process and promoted tumor metastasis [30–32], was downregulated and vimentin, fibronectin, Snail and Slug were upregulated after calponin 2 knockdown in PDAC cells. These data suggest that AKT, NF-κB pathway and the EMT process may function interrelated with the function of calponin 2 in PDAC cell proliferation and metastasis.

**Figure 7:**
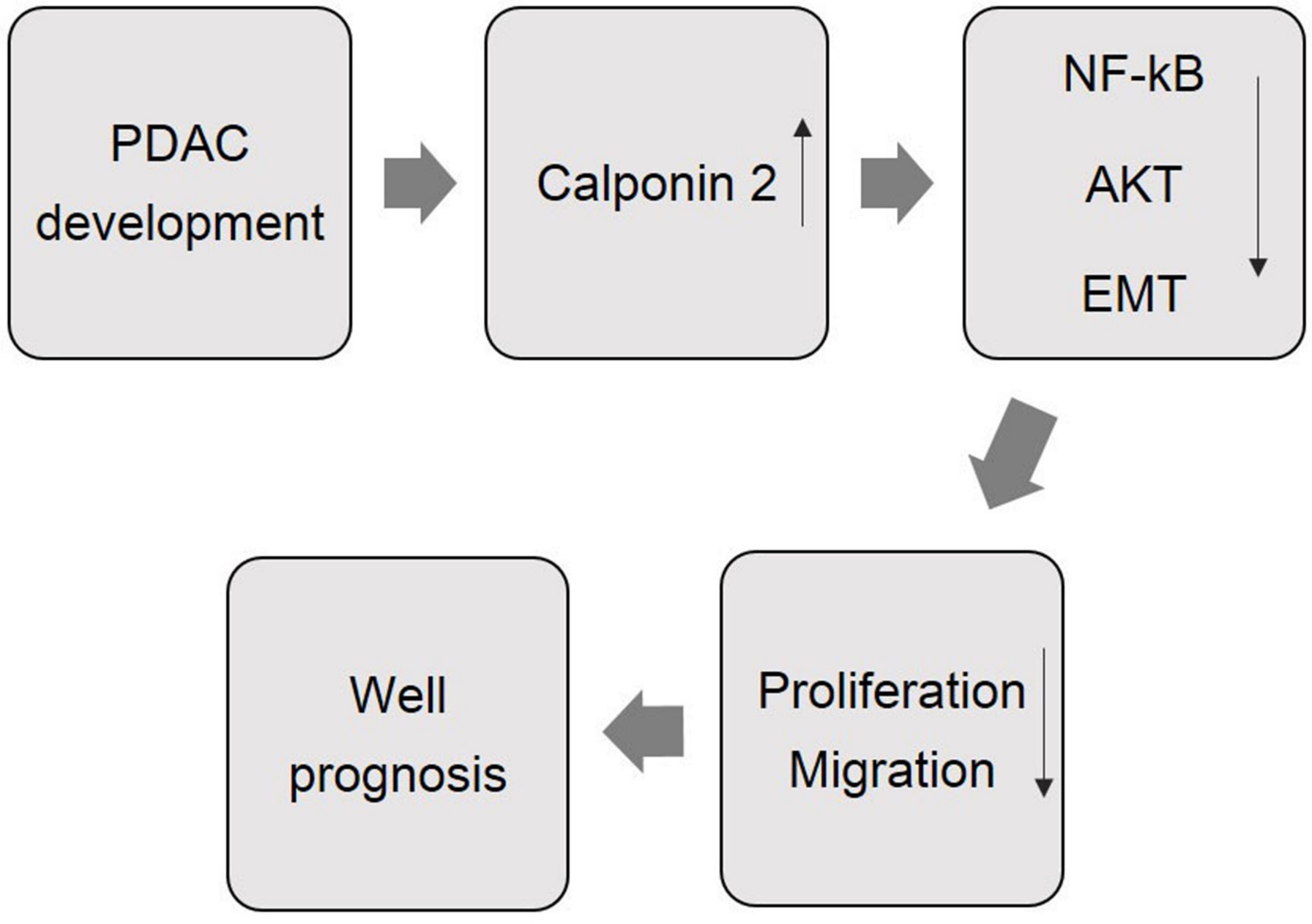
A schematic summary of the role of calponin 2 in the development and prognosis of PDAC

Since calponin 2 inhibits PDAC cell proliferation and metastasis, its expression in tumor tissue is down-regulated as previously reported in metastasis versus non-metastasis prostate cancer cells [16]. In the present study, we found that calponin 2 was not significantly expressed in normal pancreatic epithelial cells while its expression was increased in PDAC tumor cells. Therefore, the expression of calponin 2 in PDAC tumor cells may reflect epithelial-mesenchymal transition. In breast cancer cells, calponin 2 was also upregulated in comparison to that in normal breast gland cells [33]. Calponin 2 is known to function in inhibiting cell proliferation and migration. Therefore, its expression in PDAC tumor cells may act as an antagonist of migration and metastasis. The finding in PDAC is supported by previous studies of gastric cancer and acute lymphoblastic leukemia [34–37]. Our working hypothesis is that the expression level of calponin 2 in tumor cells has an inverted correlation with malignancy in terms of metastasis and prognosis (Figure 7).

Taking together, the clinical data and cellular experiments reported in the present study strongly support the notion that calponin 2 is an inhibitory modulator of tumor progression, and therefore a possible new molecular target for cancer therapy. There are remaining questions yet to be answered. Most of the PDAC patients we studied in TMA had an early stage tumor (AJCC TNM stage I and II). More patients with stage III and IV are needed to confirm our findings. Although our experiments verified the inhibitory effect of calponin 2 on cancer cell proliferation and metastasis and investigated two signaling pathways that had changes after calponin 2 knockdown, the molecular mechanism that underlies the protective regulation by calponin 2 in PDAC needs to be further elucidated.

In conclusion, we have identified calponin 2 as a novel positive prognostic factor for patients with PDAC. Higher expression of calponin 2 predicts favorable survival and correlates with less lymph node metastasis. Therefore, calponin 2 holds a promise as a molecular target in future development of a new pharmacological therapy for the treatment of PDAC.

## MATERIALS AND METHODS

### Tissue microarray

Two PDAC tissue microarrays were purchased from Outdo Biotech Co., Ltd (Shanghai, China). 119 tumor tissue spots, 99 adjacent non-tumor tissue spots, 8 positive lymph nodes and 2 normal pancreatic tissues were included. In the tissue microarrays, the diameter of each spot was 1.5 mm. The patients consisted of 78 males and 41 females 34 to 85 years of age when they underwent surgery. None of the patients have received any chemotherapy or radiotherapy before surgery. All specimens were confirmed by the Department of Pathology, Xijing Hospital using hematoxylin-eosin (HE) staining method. Clinical information, such as age, gender, tumor differentiation, lymph node metastasis and TNM stage was collected and stored in a database. All samples and clinical information were properly collected and informed consent was obtained from each patient. Among the 119 PDAC patients, 110 had complete clinical information. Furthermore, among the 110 cases, 91 had overall survival information with follow-up until death or the end date of sample collection (December, 2011). All experimental protocol were approved by Xijing Hospital Ethics Committee of the Fourth Military Medical University, which is in accordance with the NIH guidelines.

### Immunohistochemistry on TMA

Immunohistochemistry staining was performed with avidin-biotin-peroxidase method on the TMA slide using a staining kit (Zhong Shan Goldenbridge Biotech, China). Briefly, the paraffin-embedded slide were washed in xylene to remove paraffin and then followed by graduated alcohol series. Antigen retrieval was performed using citrate buffer. The slides were then incubated with 3% H_2_O_2_ for 15 minutes to inactivate endogenous peroxidase. Normal goat serum was added for 15 minutes to block nonspecific staining. Anti-calponin 2 antibody was added and incubated overnight at 4°C. The slides were washed with phosphate-buffered saline (PBS) (3 times, 5 minutes each) and incubated with biotin labeled secondary antibody for 30 minutes and horseradish peroxidase labeled streptavidin for 20 minutes, followed by DAB substrate reaction for color development. Hematoxylin was used to counter stain the nuclei. The slides were imaged under a light microscope. Brown staining cells were defined as positive expression cells. In lieu of primary antibody, only blocking serum was added for negative control.

### Assessment of immunohistochemical scoring

The immunohistochemical scoring was evaluated by two pathologists who were independently blinded for the patient information. Their readings were averaged to obtain a final score for each sample. The expression of calponin 2 was assessed based on percentage of positive cells and the intensity of staining. The percentage of positive cells was divided into five grades: score of 0 had no positive cells; score of 1 had 1–25%; score of 2 had 26–50%, score of 3 had 51–75% and score of 4 had > 75% positive cells. The intensity of staining was scored as 0 for negative, 1 for weak, 2 for intermediate, and 3 for strong staining. The overall score was the product of percentage score and intensity score. Low expression of calponin 2 was defined as overall score of 0–4 and the high expression was defined as overall score of 5–12. Each tissue was assessed in five areas and the average score was used for final analysis.

To further validate the expression of calponin 2 in tumor cells and adjacent non-tumor cells, mean densitometry calculation was also performed in 20 paired tumor tissues and adjacent non-tumor tissues using Image-Pro Plus 5.0 software. For the tumor tissues, solid tumor cellular area was selected, and for adjacent non-tumor tissues, normal gland cellular area was selected. The corresponding IOD (Integrated option density) and the cellular area was calculated by the software. Mean densitometry was calculated as IOD/cellular area. Each tissue spot was examined in 5 randomly selected high magnification field (40 × objective lens) for statistical analysis.

### Cell lines and transfection

Human pancreatic cancer cell lines MIA-PaCa2 and Panc-1 were purchased from GeneChem Co., Ltd (Shanghai, China) and cultured in DMEM supplemented with 10% fetal bovine serum (GIBCO, Carlsbad, CA, USA) at 37°C, in 5% CO_2_ atmosphere. The cell lines have been validated with their DNA profiles and compared with that of continuous cell lines available in the data bank through www.dsmz.de for authenticity. Calponin 2 overexpression lentivirus was bought from GeneChem Co., Ltd and used according to the manufacture's protocol. Small interfering RNA (siRNA) was designed by Biomics Biotechnologies Co., Ltd (Nantong, China) and transfections were performed using commercial reagent according to the manufacture's protocol (Qiagen, Germany). Three different siRNA duplexes targeting calponin 2 gene (NM___007725.2, Pubmed) were assessed to choose the most efficient one. Sequences of the siRNAs are as follows: 5′-GCACACUCAUGAACAAGCUdTdT-3′ (calponin 2 siRNA1), 5′-CGAGAAGGCAUCUCUAUGAdTdT-3′ (calponin 2 siRNA2), 5′-GGCACAUCUAUGAUACC AAdTdT-3′ (calponin 2 siRNA3). An unrelated siRNA was used as negative control.

### Cell proliferation and colony-formation assay

Cell Counting Kit-8 (Beyotime, Shanghai, China) was used to evaluate cell proliferation. 4 × 10^3^ cells per well were seeded in seven 96-well plates and cultured as above. One plate was taken out every 24 hours and 10 μl CCK-8 solution was added to each well. After 1 h incubation, absorbance at 450 nm was measured on a spectrophotometer (Thermo Scientific). For colony formation assay, 1000 cells were seeded in six-well plates and cultured for two weeks at 37°C after transfection with siRNA. At a series of time points, the cells were stained with gentian violet and imaged under a microscope using a digital camera. The experiment was repeated for three times.

### *In vitro* wound healing assay

As described previously [16], 2 × 10^5^ cells were seeded in six-well plates and cultured for 3 days. When the cells reached 80% confluence, scratch wounds were made using a sterile 200 μL pipette tip. To monitor the healing process, width of the wound was photographed and measured at a series of time points.

### RNA extraction and real-time quantitative PCR

An RNA extraction kit (Takara, Tokyo, Japan) was used to isolate total RNA of the cultured cells. The RNA sample was reverse transcribed into cDNA using a reverse transcription kit (Takara, Tokyo, Japan). The expression of calponin 2 mRNA was analyzed using real-time quantitative PCR. Calponin 2 and GAPDH specific primers were designed and synthesized by Takara. The primer sequences are as follows: calponin 2: F 5′-GAGCTCCACGCAGTTCAACA-3′, R 5′-CCTC TATCCAGCTTCGGAGTTC-3′; GAPDH: F 5′-CCTGGCCAAGGTCATCCATG-3′, R 5′-GCAGGAGACAACCTG GTCCT-3′. 20 μL reactions were examined using a Real Time Fluorescent Quantitative PCR Instrument (Bio-Rad, CA, USA) according to the manufacturer's instructions. ΔΔC_T_ was used to determine the relative expression of real-time PCR products comparing the target gene with the expression of glyceraldehyde-3-phosphate dehydrogenase (GAPDH) mRNA.

### Protein extraction and western blot analysis

Total protein was extracted with RIPA lysis buffer (Beyotime Biotechnology, Shanghai, China) and the concentration was assessed by Coomassie Brilliant Blue R250 staining. After electrophoresis in Sodium dodecyl sulfate-polyacrylamide gels (SDS-PAGE), the protein was transferred to polyvinylidene fluoride (PVDF) membranes and then incubated with anti-calponin 2 monoclonal antibody 1D2, produced at Wayne State University, USA), PCNA polyclonal antibody (Cell Signaling Technology, USA), p-Erk1/2/Erk1/2 antibody (Cell Signaling Technology, USA), p-JNK/JNK antibody (Cell Signaling Technology, USA; Abcam, England), p-p38/p38 antibody (Cell Signaling Technology, USA), p-STAT/STAT antibody (Cell Signaling Technology, USA), p-AKT/AKT antibody (Cell Signaling Technology, USA; Abcam, England), p-p65/p65 NF-κB antibody (Cell Signaling Technology, USA), E-cadherin antibody (Cell Signaling Technology, USA) and β-actin monoclonal antibody (Sigma, USA) as internal control at 4°C overnight. After washes, secondary antibodies conjugated with horseradish peroxidase (Zhong Shan Goldenbridge Biotech, China) was used for chemiluminescence (Bio-rad, CA, USA) detection of the specific proteins. Densitometry quantification of the bands was performed with Quantity One software (Bio-rad, CA, USA).

### Immunofluorescence assay

Immunofluorescence assay of cultured cells was performed as previously reported [38]. Briefly, after transfection with calponin 2-siRNA, cells were incubated on chamber slides (Corning, USA). When the cells reached a sufficient number, they were fixed in 4% paraformaldehyde for 10 minutes. Goat serum blocking solution was used before overnight incubation with anti-Ki-67 antibody (Millipore, USA), after washes, Alexa Flour 488 labeled secondary antibody (Thermo Scientific, USA) was added for 30 minutes before 4, 6-diamidino-2-phenylindole (DAPI, Roche, Switzerland) stain of the nuclei. Confocal laser scanning microscopy was performed to acquire the images.

### Cell cycle analysis and 5-Bromo-2-deoxyUridine (BrdU) incorporating assay

For cell cycle analysis, approximately 1 × 10^6^ cells were collected and fixed by 4% paraformaldehyde. Propidium iodide (Sigma, MO) was added to stain DNA and the cells were analyzed with a FACScan flow cytometer (BD Biosciences, Franklin Lakes, NJ). The results were analyzed using Multicycle-DNA Cell Cycle Analyzed Software.

For BrdU incorporating analysis, cells were cultured in 96-well plate at 2 × 10^5^ cell/ml. The BrdU incorporating analysis was performed using BrdU Cell Proliferation Assay Kit (Millipore, USA) according to the manufacturer's instructions. BrdU concentration in the sample was measured by OD_450nm_ reading.

### Statistical analysis

SPSS 19.0 software was used to perform statistical analysis (SPSS, Chicago, IL). Mann-Whitney *U* test and Chi-square test were used to compare the expression of calponin 2 between tumor tissues and adjacent non-tumor tissues. Chi-square test was performed to assess the correlation between calponin 2 expression and clinicopathological parameters. Kaplan-Meier analysis and log-rank test were used to evaluate the difference of survival between patients with high and low expression of calponin 2. Univariate and multivariate Cox regression models were carried out to test whether calponin 2 expression was an independent prognostic factor. Quantitative data of Western blot (WB) and PCR were presented as mean ± SEM and difference between groups was assessed with two-tail Student's *t*-test.

## SUPPLEMENTARY MATERIALS FIGURES AND TABLES



## Abbreviations

PDAC: pancreatic ductal adenocarcinoma
HE: hematoxylin-eosin
IHC: immunohistochemistry
PBS: phosphate-buffered saline
Si-1: calponin 2 siRNA1
Si-2: calponin 2 siRNA2
Si-3: calponin 2 siRNA3
NC: negative siRNA control
SDS-PAGE: sodium dodecyl sulfate-polyacrylamide gels
PVDF: polyvinylidene fluoride
PCNA: proliferating cell nuclear antigen
EMT: epithelial-mesenchymal transition
MAPK: mitogen-activated protein kinase
