# Supplementary material for: Increased expression of calponin 2 is a positive prognostic factor in pancreatic ductal adenocarcinoma
Source: Oncotarget. 2017 May 9;8(34):56428–42. doi: 10.18632/oncotarget.17701 (PMC5593573; doi:10.18632/oncotarget.17701)
Supplement: Supplementary file 1 [file oncotarget-08-56428-s001.pdf]

Increased expression of calponin 2 is a positive prognostic factor in pancreatic ductal adenocarcinoma

Supplementary Materials

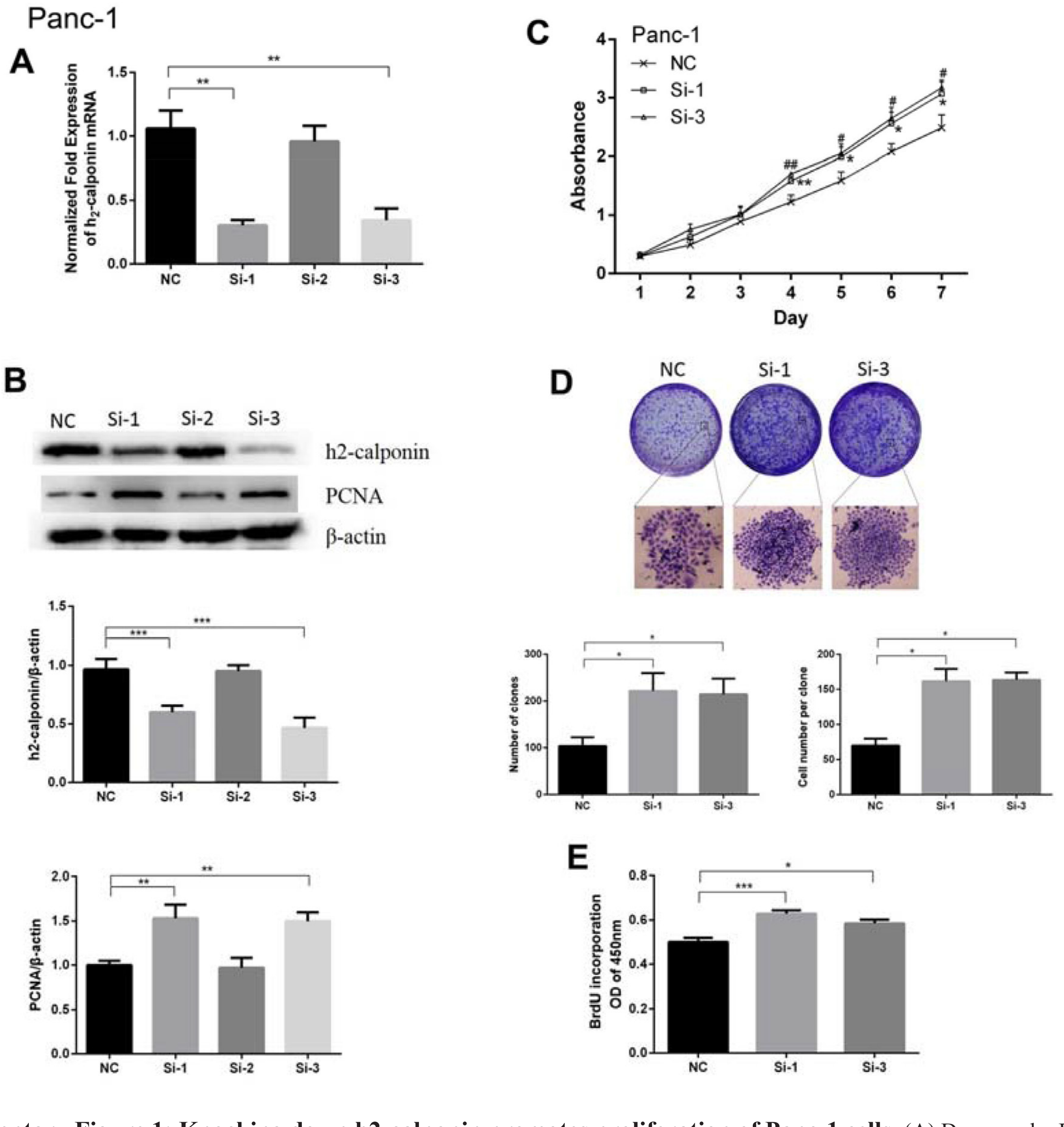

Supplementary Figure 1: Knocking down h2-calponin promotes proliferation of Panc-1 cells. (**A**) Decreased mRNA level of h2-calponin in Si-1 and Si-3 cells compared with NC ($^{**}p < 0.01$). (**B**) Decreased protein level of h2-calponin and increased protein level of PCNA in Si-1 and Si-3 cells by WB method and semiquantitative analysis ($^{**}p < 0.01$, $^{***}p < 0.001$). (**C**) Increased absorbance value and cell proliferation ability in Si-1 and Si-3 compared with NC ($^{*}p < 0.05$, $^{**}p < 0.01$, Si-1 versus NC; $^{\#}p < 0.05$, $^{\#\#}p < 0.01$, Si-3 versus NC). (**D**) Increased colony formation in Si-1 and Si-3 cells compared with NC ($^{*}p < 0.05$), magnification × 100. (**E**) BrdU assay showed that more BrdU was incorporated in Si-1 and Si-3 than that of NC ($^{*}p < 0.05$, $^{***}p < 0.001$).
